# Stock Market Liberalization and Corporate Green Innovation: Evidence from China

**DOI:** 10.3390/ijerph18073412

**Published:** 2021-03-25

**Authors:** Yuming Zhang, Juanjuan Zhang, Zhang Cheng

**Affiliations:** 1 School of Management, Shandong University, Jinan 250100, China; zhangym@sdu.edu.cn (Y.Z.); skyzhang@mail.sdu.edu.cn (J.Z.); 2 The Center for Economic Research, Shandong University, Jinan 250100, China

**Keywords:** capital market liberalization, green innovation, Shanghai-Hong Kong Stock Connect program, Shenzhen-Hong Kong Stock Connect program

## Abstract

Corporate green innovation is an effective way to achieve energy conservation and emission reduction. Enterprises’ willingness to pursue green innovation is increasingly affected by external factors. By using a quasi-natural experiment of China’s Stock Connect program, we investigate the impact of stock market liberalization on corporate green innovation. We find that stock market liberalization increases enterprises’ green innovation, especially for state-owned enterprises. We also find that stock market liberalization plays a stronger role in promoting the green invention patents of enterprises whose managers have overseas experience and enterprises in areas with a higher degree of openness. Our mechanism analysis suggests that stock market liberalization attracts the attention of securities analysts and increases managers’ focus on environmental protection, thereby promoting corporate green innovation. Our findings show that stock market liberalization plays an important role in the governance of firms’ non-financial behavior, which has important theoretical and practical implications.

## 1. Introduction

Serious environmental pollution and resource depletion pose new challenges to economic growth. Developing green innovation is a key way to achieve energy saving, pollution prevention, waste recovery, and sustainable economic growth, especially in emerging countries [1,2,3]. Research indicates that the degree of corporate green innovation is affected by factors such as government-level environmental regulations, government subsidies, and CEOs’ emphasis on environmental protection [4,5,6]. In addition to the above factors, in an open economy, the entry of foreign capital may affect enterprises’ corporate governance and development strategies [7,8,9]. Therefore, the impact of stock market liberalization on corporate green innovation in developing countries, which are shifting away from reliance on heavily polluting industries to a green economy, is particularly worthy of discussion. We explore the potential relationship between the liberalization of the stock market and corporate green innovation, asking the following important questions. Can stock market liberalization promote green innovation? If so, what is the mechanism underlying this influence? Does the liberalization of the stock market have a heterogeneous impact on the green innovation of different types of enterprises?

In the last few years, research related to green innovation-and what drives it-has been increasing rapidly [10]. At the same time, the definition of green innovation has expanded. The term “green innovation” was coined in the 1990s to refer to green technology innovation. Green technology refers to technologies, processes or products that reduce environmental pollution and the use of raw materials and energy [11]. Later, the Organization for Economic Cooperation and Development defined green innovation as hardware and software innovation related to green products [12]. For our purposes, green innovation includes energy saving, pollution prevention, waste recycling, green product design, environmental management, and other forms of technological innovation.

The literature on the factors influencing corporate green innovation has certain limitations. First, it focuses on the factors within a country. For example, some studies find that customer demand [13], relevant non-technological innovations, and the potential to gain competitive advantages can promote enterprises’ green innovation [14,15]. Second, studies in this area focus on specific means of macro-control, such as government-level environmental regulations and government subsidies; few studies focus on “non-green” control measures such as stock market liberalization [16,17,18]. Finally, research in this area pays insufficient attention to the mechanisms underlying the influence of particular factors on green innovation. Although increasing effort is being made to identify factors that affect the green innovation of enterprises, most relevant studies offer qualitative analyses that fail to consider the transmission mechanisms involved [19,20].

Clearly, the existing literature cannot effectively solve the problems raised at the beginning of this paper; we aim to fill this gap. The official launch of China’s Shanghai–Hong Kong Stock Connect and Shenzhen–Hong Kong Stock Connect programs provide a natural experimental platform for our research [8,21]. The Shanghai–Hong Kong Stock Connect program links the Shanghai Stock Exchange with the Stock Exchange of Hong Kong, allowing investors from each side to buy and sell stocks listed on the other side’s exchange within a specified scope through local securities enterprises (or brokers). Similarly, the Shenzhen–Hong Kong Stock Connect program links the Stock Exchange of Hong Kong with the Shenzhen Stock Exchange. Using China’s Stock Connect program as a natural experiment to explore the effects of stock market liberalization has the following advantages. First, under this policy, enterprises are targeted for listing in batches, creating an exogenous event similar to a “split-level” case. This helps to overcome the problem of endogeneity. Second, the Shanghai–Hong Kong Stock Connect and Shenzhen–Hong Kong Stock Connect programs give foreign investors opportunities to invest in China’s stock market. This allows observers to more easily and intuitively detect changes in China’s stock market liberalization at a given point, avoiding measurement error relating to the degree of liberalization [22]. Finally, two kinds of “natural” stocks are listed on the Stock Exchange, providing a natural experimental group and a control group for our model. In addition, the Stock Connect program provides a homogeneous information environment [23]. Taking listed enterprises in the same country as the research sample, we can control for the impact of other economic policies on the efficiency of the capital market. This can mitigate potential bias in the research results caused by institutional factors and missing variables.

We use the quasi-natural experiment of China’s stock market liberalization, as represented by the Stock Connect program. The treated group comprises enterprises participating in the Shanghai–Hong Kong Stock Connect program or the Shenzhen–Hong Kong Stock Connect program, and the control group includes enterprises that are not eligible to trade via either channel. Based on data covering the 10 years from 2010 to 2019, we used a time-varying difference in differences (time-varying DID) model to empirically test the impact of stock market liberalization on corporate green innovation and the mechanism underlying the transmission of influence. The results indicate that the liberalization of the stock market increases the degree of green innovation of listed enterprises. In total, two transmission mechanisms enable stock market liberalization to promote green innovation. First, liberalizing the stock market can improve the environmental awareness of enterprise managers, leading them to pay more attention to environmental protection and green development in corporate governance. This, in turn, can increase corporate green innovation. Second, liberalizing the stock market can attract securities analysts’ attention, which reduces information asymmetry, and thus increases corporate green innovation. In addition, we carry out a series of robustness tests to ensure the robustness of the regression results. Finally, we find that stock market liberalization has a heterogeneous impact on enterprises’ green innovation, varying by enterprise type.

Our main contributions are as follows: first, we enrich and expand the literature on the factors influencing corporate green innovation. Most studies in this area focus on the influence of government-level environmental regulations, government subsidies, and CEOs’ attention to environmental protection [24,25,26]. Few consider how corporate green innovation is influenced by policies other than designated “green” policies. We explore the impact of stock market liberalization on corporate green innovation and the mechanisms underlying this influence, contributing to theory and expanding the research in related fields. Second, based on information asymmetry theory, we conduct a detailed mechanism analysis of how the capital market affects corporate green innovation. Numerous studies show that stock market liberalization can strengthen corporate governance mechanisms, and reduce information asymmetry [27,28]. For example, stock market liberalization enhances the mechanism of supervision of enterprise management, improves the information environment, and strengthens the supervision of enterprises’ innovation behavior [29,30]. We extend information asymmetry theory to enterprise green development, opening up a new avenue for future research. Finally, we demonstrate that liberalizing the stock market significantly increases enterprises’ green innovation. This finding fills a gap in stock market liberalization research by linking stock market liberalization with enterprises’ clean development.

## 2. Background and Hypotheses

### 2.1. Background

Together, the Shanghai–Hong Kong Stock Connect and Shenzhen–Hong Kong Stock Connect programs are a mechanism connecting stock market transactions between Shanghai, Shenzhen, and Hong Kong. The Shanghai–Hong Kong Stock Connect program was officially launched in 2014. Initially, the program included the stocks making up the SSE (Shanghai Stock Exchange) 180 Index and the SSE 380 Index, as well as the stocks of enterprises cross-listed on the Shanghai Stock Exchange and Hong Kong Stock Exchange. Since 2014, the number of underlying stocks eligible for trading via the Shanghai–Hong Kong Stock Connect has continuously increased, in turn increasing the scale of the program. Given the smooth operation of the Shanghai–Hong Kong program and building on the experience accumulated, the Shenzhen–Hong Kong Stock Connect was officially launched in 2016. The first batch of stocks eligible for trading via the Shenzhen–Hong Kong Stock Connect included those making up the SZSE (Shenzhen Stock Exchange) Stock Index and the SZSE Small/Mid Cap Innovation Index, as well as the stocks of enterprises crossed-listed on the Shenzhen Stock Exchange and Hong Kong Stock Exchange. Since 2016, the number of participating enterprises has continuously increased, expanding the program’s stock range. The Stock Connect program has accelerated the liberalization and gradually relaxed the supervision of China’s stock market, enabling foreign investors to enter China through Hong Kong.

The implementation of this policy was a landmark event in the liberalization of China’s stock market, opening it up to the outside world. The two programs have not only widened the investment opportunities for investors in mainland China and Hong Kong, but also enriched the investor structure of the A-share capital market, which has far-reaching significance for China’s local stock market [31]. Meanwhile, as a developing country, China has paid growing attention to environmental protection in recent years. Chinese enterprises are increasingly engaging in green innovation, and the demand for green innovation is gradually rising. China’s stock market liberalization provides us with a natural experimental group and control group for in-depth empirical analysis of the impact of stock market liberalization on corporate green innovation.

### 2.2. Theoretical Background

According to information asymmetry theory, those involved in market economic activities differ in the amount of information they possess. Parties with sufficient information occupy favorable positions in the market, while parties with insufficient information are at a disadvantage [32,33,34]. Information asymmetry encourages parties who possess sufficient information to further their own interests at the expense of parties that lack information, thereby triggering moral hazard and adverse selection problems. This kind of information asymmetry generally exists between listed companies and shareholders [35]. Connecting capital markets can reduce information asymmetry. China’s Stock Connect program has drawn global attention to the participating companies, not only from foreign investors but also from media outlets, analysts, and auditing agencies. This has greatly increased the participating companies’ information transparency, reduced information asymmetry between companies and investors, and improved the information environment [36]. The attention of mature foreign investors and institutional bodies provides international supervision for the participating enterprises, limiting managers’ incentives to engage in self-interested behavior [37]. This in turn encourages managers to formulate and implement environmental protection and green development strategies, ultimately increasing companies’ engagement in green innovation.

### 2.3. Hypotheses

#### 2.3.1. Positive Perspective

Stock market liberalization in developing countries has attracted many international investors from developed regions. Based on the theory of information asymmetry, as outlined above, the increased presence of foreign investors increases corporate information transparency and improves corporate governance, in turn promoting corporate green innovation. On the one hand, stock market liberalization allows domestic companies to compete in the global capital market and attracts foreign investment on a global scale. To attract foreign investment, enterprise managers must acknowledge the importance of environmental protection and take the initiative in improving corporate governance and green innovation. At the same time, managers can also learn about environmental protection from mature foreign investors, thus enhance their environmental awareness and actively carry out green innovation [38]. On the other hand, by strengthening supervision, the entry of foreign investors forces enterprises to pay more attention to long-term development, encouraging them to reduce their speculation, and establish green and sustainable development strategies [39]. Next, we conduct a more detailed analysis of the two pathways by which liberalizing the stock market promotes green innovation.

First, liberalizing the stock market can increase the environmental awareness of managers and thus promote corporate green innovation. Compared with those in developing countries, institutional investors from developed regions often have a more mature investment knowledge and a greater awareness of the importance of environmental protection, and they pay more attention to the green and sustainable development of enterprises. For example, using data from the Standard and Poor’s 500 index, which represents stocks from developed regions, Jagannathan et al. (2017) find that managers in developed countries tend to incorporate environmental criteria into their investment decisions [40]. Wen (2009) finds that institutional investors in Anglo-American countries prefer to invest in enterprises that display a sense of social responsibility, including responsibility for environmental protection [41]. Therefore, liberalizing the stock market raises managers’ environmental awareness in the following two ways: on the one hand, opening the capital market can increase the proportion of foreign investors among shareholders. To protect their own interests and avoid potential risks associated with information asymmetry, they may intervene considerably in corporate governance. Cuervo et al. (2002) find that, in both large shareholder oriented and market-oriented systems, institutional investors participate in corporate governance to maximize corporate value [42]. Abdioglu et al. (2013) find that governance quality in a foreign institutional investor’s home country can determine the investor’s investment decisions. When the quality of governance in the investor’s home country is high, the investor will display stronger enterprise governance behavior [43]. When foreign investors engage in corporate governance, their environmental protection knowledge may influence the corporate culture, raising managers’ environmental awareness, and encouraging them to establish green development strategies. On the other hand, to attract foreign investors, the manager will strive to cater to their preferences [44,45]. When managers realize that environmentally friendly corporate practices are popular among foreign investors, they will take the initiative in raising environmental awareness.

Managers with a high level of environmental awareness are likely to promote corporate green innovation significantly. Green innovation requires a large investment and has a low success rate. The greater a managers’ environmental awareness is, the better equipped they are to identify the potential benefits and market opportunities of green innovation [46,47], thereby increasing the success of their enterprises’ green innovation. In addition, managers with greater environmental awareness pay more attention to the sustainable development of their enterprises. They can more effectively curb the implementation of highly polluting activities and actively engage in green innovation, assisting in the development of environmentally friendly enterprises [48,49]. In sum, liberalizing the stock market can increase managers’ environmental awareness, thereby enhancing corporate green innovation.

Second, liberalizing a country’s stock market strengthens the external supervision of domestic companies, ultimately increasing their engagement in green innovation. Stock market liberalization can attract the attention of external stakeholders of a company, leading to more rigorous supervision by audit institutions, analysts, media outlets, etc. For example, Bae et al. (2006) document the stock market opening process in emerging stock markets, and find that increased openness can increase analyst coverage and the availability of firm-specific information [50]. Fang et al. (2015) conclude that mature investors from developed countries can promote the global convergence of financial reporting practices [51]. Tsang et al. (2019) find that foreign institutional ownership leads to a greater increase in firms’ voluntary disclosures than domestic institutional ownership [52]. According to the theory of information asymmetry, foreign investors have certain information disadvantages compared with corporate managers. The more attention a company receives from external stakeholders such as securities analysts, the greater its information transparency and the better its information environment [50,53]. Therefore, the increase in company information disclosure caused by the opening of the capital market alleviates information asymmetry, which to some extent can restrain inappropriate investment behaviors caused by principal-agent problems. For example, Bae et al. (2012) find that the increased presence of foreign investors accelerates the transmission of market information, thereby reducing price delay and information asymmetry between investors and enterprise management [30]. Kim et al. (2016) find that foreign institutions’ monitoring effectiveness improves when the gap in monitoring technology between foreign and domestic institutions widens [54]. These findings suggest that foreign investors can provide effective supervision of corporate governance behavior. When foreign investors pay more attention to the green and sustainable development of enterprises, with a corresponding increase in corporate information transparency, corporate environmental pollution behavior will be more susceptible to supervision and restraint, and companies will engage in more green innovation. We can infer that after stock market liberalization, the number of analysts tracking listed companies participating in the program will increase, which, in turn, will encourage these companies to carry out green innovation.

Based on the above analysis, we propose the following hypothesis:

**Hypothesis** **1** **(H1).** *Stock market liberalization promotes the green innovation of enterprises.*

#### 2.3.2. Negative Perspective

However, changes to the stock market environment brought by the liberalization of the stock market may also inhibit the green innovation of enterprises.

First, although liberalizing the stock market ultimately strengthens the supervision of enterprises and reduces information asymmetry, realizing these benefits takes time and comes at a cost [55]. In the short term, foreign investors are still somewhat restricted in their access to enterprise information [56]. For example, such information may only be accessible via site visits and analysts’ tracking reports. For foreign investors, this kind of supervision requires a lot of time and energy, potentially reducing its effectiveness. Compared with green innovation, which has long-term value, indicators that reflect the degree of enterprise development in the short term, such as return on assets (ROA) and return on equity (ROE) are easier to observe and assess [57]. To prevent the outflow of foreign capital, enterprises may focus on improving their short-term performance, which is relatively easy to observe, and thus reduce their investment in green innovation projects.

Second, liberalizing the stock market may increase stock volatility and macroeconomic instability [58]. This will increase the risk awareness of managers and enterprises, discouraging investment in corporate green innovation. In addition, the financial contagion effect caused by the liberalization of the stock market will increase the risk of corporate bankruptcy and mergers [59]. As a result, managers may experience a degree of psychological resistance to innovation behavior due to its high level of uncertainty. If a corporate green innovation project fails, the news will quickly spread to external investors, which may lead to the outflow of foreign capital and disrupt internal governance. Therefore, in the interests of their own development, enterprises may choose to focus on stable and profitable production activities and reduce their engagement in green innovation projects.

Finally, as the participation of foreign investors strengthens the supervision of managers, it may put greater pressure on enterprises to replace their managers [60,61]. This, in turn, will incentive managers to pursue stable approaches to governance and weaken their motivation to engage in green innovation. The liberalization of the stock market reduces information asymmetry between enterprises and investors, making it easier for foreign investors to participate in corporate governance. However, green innovation is costly, takes a long time to implement and generates uncertain financial rewards, which may dissuade managers from pursuing green innovation [62]. In sum, the presence of foreign investors may increase the pressure on managers to perform well in the short term, intensify their short-sighted behavior and reduce their engagement in high-risk green innovation activities that enhance the long-term value of the enterprise.

Based on the above analysis, we propose the following opposing hypothesis:

**Hypothesis** **2** **(H2).** *Stock market liberalization restrains the green innovation of enterprises.*

## 3. Methodology

### 3.1. Data

As China faces severe environmental pollution and resource depletion, green innovation is receiving increasing attention. The process of stock market liberalization in Shanghai and Shenzhen in 2014 and 2016, respectively, provides a good source of data. The data of Chinese listed enterprises in Shanghai and Shenzhen stock market from 2010 to 2019 are used in this paper. Following Pan et al. (2020) and Ren et al. (2020), we use the number of green patents to measure an enterprise’s green innovation [63,64]. The patent data are collected manually from the State Intellectual Property Office of the People’s Republic of China, which is the most authoritative source of statistical data on patent applications and authorizations in China. In 2010, the World Intellectual Property Organization (WIPO) launched an online tool designed to facilitate searches for patent information relating to environmentally sound technologies, known as the “International Patent Classification (IPC) Green Inventory”. The search items in this inventory classify green patents into seven major categories according to the United Nations Framework Convention on Climate Change: waste management, energy conservation, transportation, alternative energy production, administrative regulatory or design aspects, agriculture or forestry, and nuclear power generation. According to the “IPC Green Inventory” launched by WIPO, we calculate the annual numbers of green invention patents and green utility model patents and use them as the core index to measure the green innovation activities of enterprises.

Other major economic data are from the China Stock Market and Accounting Research database. This database is supported by the national government and many scientific research units and contains the most accurate and comprehensive information on Chinese listed enterprises. We ignore data on enterprises in the financial industry and A+H share enterprises (enterprises cross-listed on one of the mainland Chinese Stock Exchanges and Hong Kong Stock Exchange). To eliminate the influence of repeated values and missing values on the regression results, we screen the data. To eliminate the influence of outliers and increase the reliability of the data, we carry out 1% bilateral indentation processing for continuous variables. We collect data from the Wind database to replace missing data, and use STATA14 (StataCorp LLC, College Station, TX, USA) for the statistical analysis.

### 3.2. Variables

We select the number of green invention patent applications (greinnov1) and green utility model patent applications (greinnov2) of the listed enterprises as our analysis objects. We use the number of green patent applications, not the number of green patents authorized, to measure green innovation. Research shows that patents have already impacted the performance of enterprises at this stage of the application. The process of applying is less affected by bureaucratic factors than the patent authorized. Therefore, data on patent applications are generally more stable, timely, and reliable than authorization data [65]. Patent applications also reflect enterprises’ degree of emphasis on innovation, which are in line with our research topic.

The independent variable selected in this paper is a dummy variable (SHHK). It indicates whether the enterprise’s stock is in the list of the Stock Connect program in the year *t*. If an enterprise is in the list of the Shanghai or Shenzhen Stock Connect program in the year *t*, SHHK equals 0 before the year *t*, and 1 after the year *t*. If an enterprise’s stock is not in the list of the Shanghai or Shenzhen Stock Connect program, it has a value of 0 in all years. This variable is widely used by researches because the number of shares purchased by foreign investors is not disclosed [66,67].

Following previous studies [63,64], we control for a set of factors from the corporate operational and financial characteristics, including the sustainable growth rate (sustainable), education background of CEO (education), intangible assets (intangible), non-current assets (ncurrent), return on assets (ROA), enterprise size (size), Tobin’s Q value (Tobin’s Q), government subsidies amount (subsidies), financial leverage (leverage), Net cash generated by the operations (cash), shareholding ratio of the board of supervisors (supervisor), total profit (profit), net profit (netpro). In addition, we controlled the industry fixed effects (Industry FE), firm fixed effects (Firm FE), and time fixed effects (Time FE). The specifications of all these variables are described in Appendix A.

### 3.3. Models

To investigate the impact of stock market liberalization on the green innovation of enterprises, we construct a Time-varying Difference in Differences (Time-varying DID) model, as follows:

(1) $$Green\;innovation_{it}=\beta_{0}+\beta_{1}SHHK_{it}+\beta_{j}Controls_{it}+Industry\;FE+Time\;FE+Firm\;FE+\varepsilon_{it}$$

In Equation (1), the subscript *t* represents time and *i* represents enterprise. *Green innovation* refers to the number of green invention patent applications and green utility model patent applications by enterprise *i* in year *t*. *SHHK* is the independent variable. If an enterprise’s stock is listed in the Stock Connect program, it equals 0 before year *t* and 1 after year *t*. If an enterprise’s stock is not listed in the Stock Connect program, it has a value of 0 in all years. *Controls* represents the control variables, which measure other factors that may affect corporate green innovation, such as enterprise size, return on total assets and government subsidies. *Industry FE* is the industry fixed effect, *Time FE* is the time fixed effect, *Firm FE* is the firm fixed effect and $\varepsilon_{it}$ is the error term. The estimation coefficient $\beta_{1}$ is of special interest in this paper, which captures the impact of stock market liberalization on the green innovation of enterprises.

### 3.4. Summary Statistics

The descriptive statistics on the main variables are shown in Table 1.

Summary statistics are presented in Table 1. As shown in Panel A, the minimum number of green invention patent applications and green utility model patent applications is 0; the maximum numbers are 250 and 109, respectively; the mean numbers are 1.55 and 0.91, respectively. This indicates that there are great differences in green innovation between the enterprises, but that on the whole, the enterprises engage in little green innovation. The descriptive statistics for the control variables are also in line with expectations: the average value of Tobin’s Q is 1.91, the average value of return on total assets is 0.05, and the average value of leverage is 0.40. The statistical results for the other variables are similar to those reported in previous studies, so the details are not discussed here. As shown in Panel B, the total number and average number of applications for green innovation patents are gradually increasing, suggesting that enterprises are paying growing attention to green innovation.

## 4. Empirical Results

### 4.1. Preliminary Regression Results

We first test the direct effects of stock market liberalization on the green innovation of enterprises. The regression results for the direct effects in this paper are shown in Table 2. In Columns (1) and (2), only the independent variables are included, and the coefficients of the term SHHK are significant and positive (*β* = 3.586, *p* < 1%; *β* = 1.042, *p* < 1%). In Columns (3) and (4), we introduce the control variables to examine the effect of participating in the Stock Connect program on green innovation. The coefficients of this term are significant and positive (*β* = 2.493, *p* < 1%; *β* = 0.578, *p* < 5%), which suggest that after the implementation of stock market liberalization, enterprises whose stocks can be traded by foreign investors are likely to engage in more green innovation. Therefore, Hypothesis 1 is supported.

Regarding the regression results for the control variables, the coefficients of leverage and sustainable growth rate are significantly negative, these indicate that, with an increase in the sustainable growth rate and corporate leverage, enterprises’ green innovation decreases. The coefficients of ROA and government subsidies are significantly positive, indicating that enterprises that have a higher ROA or receive more government subsidies engage in more green innovation. The regression coefficients of the other variables are also generally consistent with those reported in existing literature, and thus are not discussed in this paper.

### 4.2. Mechanism Analysis

These results suggest that stock market liberalization can promote enterprises’ green innovation. Based on the foregoing theoretical analysis, we conduct preliminary empirical tests of the potential mechanism of this influence. We examine this mechanism from two perspectives: managers’ environmental awareness and analysts’ attention to enterprises.

We conduct textual analysis to measure managers’ awareness of environmental protection, focusing on the “Board Report” section of enterprises’ annual reports [68]. This process has four steps, as follows. First, we choose 76 keywords related to green environmental protection, such as “green,” “environmental protection”, and “emission reduction,” identified by searching the literature on green environmental protection and consulting Chinese dictionaries. Second, we remove unsuitable keywords, i.e., words and phrases that appear at a low frequency and are thus on the periphery of cognition. We crawl all of the enterprises’ Board Report documents using Python, analyze the documents using the software package ROSTCM 6.0 (Wuhan University, Wuhan, China), and, finally, remove 10 keywords that appear fewer than five times.

Based on the previous set of keywords, the total frequency of all keywords in year *t* for each enterprise is calculated as a proxy for managers’ green awareness. We draw on Judd et al. (1981) and Baron et al. (1986) to construct the following test equation [69,70]:

(2) $$Green\;innovation_{it}=\beta_{0}+\beta_{1}SHHK_{it}+\beta_{j}Controls_{it}+Industry\;FE+Time\;FE+Firm\;FE+\varepsilon_{it}$$

(3) $$M_{it}=\beta_{0}+\beta_{1}SHHK_{it}+\beta_{j}Controls_{it}+Industry\;FE+Time\;FE+Firm\;FE+\varepsilon_{it}$$

(4) $$Green\;innovation_{it}=\beta_{0}+\beta_{1}SHHK_{it}+\beta_{2}M_{it}+\beta_{j}Controls_{it}+Industry\;FE+Time\;FE+Firm\;FE+\varepsilon_{it}$$

*M* is the intermediary variable, which represents managers’ attention to environmental protection and analysts’ attention to enterprises. As the regression test of Formula (2) is reported in Table 2, it is not repeated here. Below we report regression analyses of Formulas (3) and (4).

In Table 3, Column (1) shows the results of regressing stock market liberalization on managers’ attention (ceoatt) to environmental protection. The regression coefficient of SHHK is 2.215, which is significant at the level of 1%. This result shows that the managers have increased their attention to environmental protection after the enterprise participating in stock market liberalization. Column (2) and Column (3) show the regression results for enterprises’ green innovation in relation to SHHK and ceoatt, the results in Column (2) show that the coefficient of SHHK and ceoatt are both significantly positive at the level of 1%, and the results in Column (3) show that the coefficient of SHHK and ceoatt are both significantly positive at the levels of 5 and 1%, respectively. These results show that the connect of capital market can promote the green innovation of enterprises by drawing managers’ attention to green environmental protection.

Column (4) shows the results of regressing stock market liberalization on analysts’ attention (anaatt). The regression coefficient of SHHK is 0.884, which is marginally significant. This suggests that connect of the capital market attracts analysts’ attention to the enterprises participating in stock market liberalization. Column (5) and Column (6) show the regression results for enterprises’ green innovation in relation to SHHK and anaatt. The results in Column (5) show that the coefficients of SHHK and anaatt are significantly positive at the levels of 5 and 1%, respectively. The results in Column (6) show that the coefficients of SHHK and ceoatt are both significantly positive at the 5% level. These results show that the connect of capital market can attract analysts’ attention, reduce information asymmetry and increase enterprises’ information transparency, all of which can encourage enterprises to engage in green innovation. The above results verify the accuracy of the theoretical analysis of this paper.

### 4.3. Robustness Tests

In addition, to increase the reliability of our findings, we perform some robustness tests of the main results.

**Figure 1:**
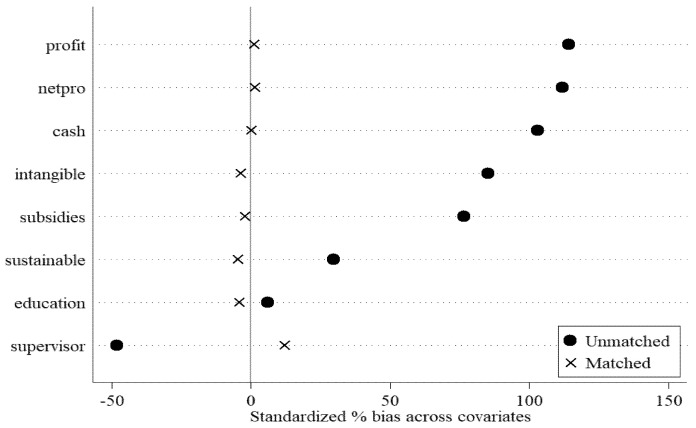
Standardized bias.

First, we use the propensity score matching difference in differences (PSM-DID) method. To accurately assess the impact of stock market liberalization on the green innovation of enterprises, it would be ideal to compare the green innovation of the same enterprise before and after stock market liberalization. However, such “counter factual” outcomes cannot be observed in reality. Therefore, to mitigate potential selection bias, we use the PSM-DID method for analysis. We take the control variables related to green innovation as matching variables. After 1:2 nearest neighbor matching with a 0.05 caliper, we remove samples that have not been successfully matched and then perform the DID model again. The balance tests and regression results are presented in Table 4. As shown in Panel A, the sample is balanced after matching. Figure 1 shows more intuitively that the standardized biases of most variables are reduced after matching. After the PSM-DID estimation, as shown in Panel B, the coefficients of SHHK are still significantly positive (2.305 and 0.581, respectively). This indicates the robustness of the positive effect of stock market liberalization on corporate green innovation.

Second, we extend the sample observations in 2014 and 2016. Take into account that the Shanghai–Hong Kong Stock Connect program and the Shenzhen–Hong Kong Stock Connect program were launched in November and December, respectively, we restore the sample observations for 2014 and 2016, and then regard 2014 and 2016 as the years in which the policy was not implemented. We conduct the regressions again. The conclusion is basically consistent. The specific regression results are shown in Column (1) and Column (2) in Table 5.

Third, we conduct a placebo test. There may be a pseudo regression problem in the correlation between stock market liberalization and corporate green innovation. Therefore, we further verify the results using a placebo test, assuming that the stock market liberalization event occurred in 2013. The coefficient of SHHK1 is no longer significant, which indicates that our results are not caused by differences in the inherent characteristics of the sample enterprises in the treated group and the control group. This supports the conclusion of this paper. The specific regression results are shown in Column (3) and Column (4) in Table 5.

Finally, the important assumption of the DID model is that the trends of the control group and treated group are similar before the policy occurred. Therefore, according to Beck et al. (2010) [71], we evaluate the parallel trends. We use the following model:

(5) $$Green\;innovation_{it}=\alpha +\sum_{j=2}^{4}\beta_{j}before_{ij}+\sum_{k=1}^{3}\gamma_{k}after_{ik}+\delta Controls_{it}+Industry\;FE+Time\;FE+Firm\;FE+\varepsilon_{it}$$

In Formula (4), *before* is a series of dummy variables. If the enterprise is in the list of the Shanghai or Shenzhen Stock Connect program in the year *t*, it equals 1 in the *j*th year before the year *t*, and 0 otherwise. Similarly, *after* equals 1 in the *k*th year after the enterprise participating in the Shanghai or Shenzhen Stock Connect program, and 0 otherwise. If an enterprise’s stock is not in the list of the Stock Connect program, it has a value of 0 in all years. All the coefficients of *β* should be insignificant if the parallel trend assumption is satisfied.

We set the year before the enterprise participates in the Stock Connect program as the base year. The data in the base year are omitted so that the multicollinearity is avoided [72]. According to Table 6, all the coefficients of *γ* (after1 to after3) are significant, while the coefficients of *β* (before4 to before2) are not. This suggests that treated and control groups followed similar trends before the Stock Connect program, and the difference between the trends in these two groups began to diverge after the Stock Connect program.

## 5. Cross-Section Analysis

### 5.1. Corporate Manager Heterogeneity

Different types of managers have different approaches to governance and different business styles. We further explore the effects of stock market liberalization between heterogeneous managers. Managers’ overseas experience in the emerging country has an important impact on their social responsibility behaviors, such as environmental protection [73]. The power of managers also affects companies’ approach to corporate governance [74]. We add the dummy variable overseaex, duality, the cross term of overseaex and SHHK, and the cross term of duality and SHHK to the original regression model. The results are shown in Table 7. The results show that the regression coefficient of cross items in Column (1) is significantly positive. It indicates that when the enterprise manager has overseas experience, the increase in their green invention patents is more obvious after the connection of capital market, and there is no significant difference for green utility model patents. The regression coefficient of cross items in Column (4) is positive and marginally significant, indicating that the increase in green utility model patents of enterprises with more managerial power is more obvious after the connect of the capital market. There is no significant difference for corporate green invention patents.

### 5.2. Enterprise Heterogeneity

As different types of enterprises differ in their governance ability and operation mode, it is necessary to further explore the differences between heterogeneous enterprises. According to the nature of enterprise property rights, we add the dummy variable SOE (If the enterprise is a state-owned enterprise, the variable is assigned to 1; otherwise, the variable is assigned to 0) and the cross term of SOE and SHHK to the original regression model to better understand the relationship between stock market liberalization and enterprise green innovation. The results are shown in Column (1) and Column (2) in Table 8. The results indicate that the coefficients of the cross terms are positive and marginally significant, which indicates that the increase in the green innovation of state-owned enterprises is more obvious after the connection of capital market. Due to their unique political and economic background, state-owned enterprises may be more likely to be sheltered by the government and exhibit less environmental protection behavior before stock market liberalization. After the connection of the capital market, however, the information transparency of state-owned enterprises increases, which has a strong governance effect on their behavior, leading them to engage in more green innovation. In contrast, as non-state-owned enterprises have already engaged in more green innovation, their behavior is less affected by the connection of the capital market.

### 5.3. Regional Openness Heterogeneity

To measure the impact of regional openness, we use data from the “China Opening-up Index” published by the International Cooperation Center of China National Development and Reform Commission to group the corresponding regions. According to these data, the degree of regional opening up is divided into four levels. The greater the degree of opening up, the higher the level is. Specifically, we add the dummy variable openness and the cross term of openness and SHHK to the original regression model, and the results are shown in Columns (3) and (4) in Table 8. The results show that the regression coefficient of the cross items to green invention patents is 3.071 and marginally significant. It indicates that after the connection of capital market, the increase in green invention patents of enterprises in regions with a higher degree of opening up is more obvious. There is no significant difference in the influence of regional openness on the relationship between capital market openness and corporate green utility model patents.

## 6. Conclusions

### 6.1. Key Findings

The problem of environmental pollution in developing countries is receiving increasing attention worldwide. Due to imperfections in the capital markets of developing countries, investors in these countries may show more irrational behaviors, making it difficult to accurately identify the value of green innovation. In contrast, in developed capital markets, many institutional investors have high-quality information collection, interpretation and analysis skills. They can obtain internal value information on enterprises, especially non-financial information, and carry out market transactions based on that non-financial information. With the opening of the world economy, investors and enterprises in developing countries may be affected by international investors. Using the exogenous event of China’s stock market liberalization, we use a Time-varying DID model to investigate the impact of stock market liberalization on the green innovation of enterprises in developing countries. We find that stock market liberalization significantly improves the green innovation of enterprises. Improving managers’ environmental awareness and attracting analysts’ attention to enterprises are important pathways for the influence of stock market liberalization on corporate green innovation. Further analysis reveals that the promotion effect is particularly strong for state-owned enterprises. Stock market liberalization has a more significant positive effect on promoting green invention patents for enterprises whose managers have overseas experience and for enterprises in regions with greater openness. Meanwhile, stock market liberalization plays a greater role in promoting green utility model patents for enterprises with more managerial power.

### 6.2. Theoretical Contributions

We make two main theoretical contributions. First, our findings extend research on corporate green innovation by investigating the effect of stock market liberalization on corporate green innovation. Studies of the factors influencing corporate green innovation focus mainly on environmental protection and energy policies, market turbulence, manager characteristics, and so on [25,26,75]; they pay little attention to the influence of “non-green” policies. We thus enrich theory and supplement the literature on the factors influencing corporate green innovation, suggesting a direction for follow-up research.

Our second contribution lies in our theoretical framework for examining how stock market liberalization can improve corporate green innovation. Studies show that stock market liberalization enhances the supervision of enterprise management, improves the information environment and strengthens the supervision of corporate innovation [27]. Based on information asymmetry theory [32,33,34], our results further reveal that stock market liberalization can increase managers’ environmental awareness and attract more attention from analysts, thus increasing enterprises’ green innovation. Our study sheds light on the mechanism by which stock market liberalization improves corporate green innovation. This enriches the literature on the impact of stock market liberalization on corporate innovation, corporate governance and corporate cleaner development, opening up a new pathway for research on stock market liberalization.

### 6.3. Implications

The findings of this paper have important practical implications. First, against the backdrop of serious environmental pollution and resource depletion worldwide, our results indicate the importance of stock market liberalization to corporate green governance, especially in emerging countries. In emerging markets such as China, connecting capital markets can significantly promote the green innovation of enterprises by allowing more mature investors to intervene in the governance of enterprises. To effectively control environmental pollution and foster a green economy, China should also further improve the stock market liberalization mechanism and actively seek foreign capital. The liberalization policies will help to establish a more rational and effective capital market through a more diversified market mix and investor pool, thereby promoting the cleaner development of the global economy.

Second, the managers’ focus on environmental protection and analysts’ attention can significantly affect the environmental governance behavior of enterprises. Therefore, we should further strengthen and improve capital markets, especially in developing countries. We should reduce enterprises’ information asymmetry, strengthen the supervision and inspection of enterprise management. We should also clarify the penalties for contravening environmental protection laws and regulations, and establish preferential policies and other means to improve managers’ environmental protection awareness and enthusiasm for engaging in enterprise environmental governance.

Finally, enterprises—especially state-owned enterprises—are expected to face increasingly serious problems associated with environmental pollution in the processes of production and operation. Therefore, enterprises should actively take responsibility for environmental protection, formulate and improve sustainable development strategies, strictly abide by environmental protection policies and measures, and enhance their environmental governance capacity. In developing countries, it is particularly important for managers with non-overseas experience to become more environmentally aware and seek to attract foreign investment. Besides, the government should strengthen its supervision of enterprises in less open areas. To maximize the contribution of foreign capital to environmental governance in developing countries, we urge enterprises in such countries to make efficient use of foreign capital to accelerate green innovation, speed up R&D to generate new products, and thus enhance their competitive advantage. Our results provide evidence of the governance effects of stock market liberalization policies in emerging markets such as China.

### 6.4. Limitations and Further Research

This study has several limitations. First, our data are limited to China. Evidence from other countries would help to assess the generalizability of the results. Future research could consider the impact of stock market liberalization on corporate green innovation for non-Chinese firms. Second, our data are limited to listed companies; we can collect data from non-listed companies to see the changes in the results. Finally, because data on foreign investors’ investment amounts and target firms are not accessible, similar to the existing research [7], we only consider whether a firm is in the list of the stock market liberalization. When relevant data become available in the future, more accurate variables can be used to measure the treated group, and better empirical evidence for verifying the results can be offered.

## Figures and Tables

**Table 1:** Descriptive statistics.

**Panel A. Descriptive Statistics of Related Variables**					
**Variables**	**N**	**Mean**	**sd**	**min**	**max**
greinnov1	4086	1.55	11.12	0.00	250.00
greinnov2	4086	0.91	4.50	0.00	109.00
SHHK	4086	0.22	0.41	0.00	1.00
education	4086	3.69	0.75	1.00	4.00
sustainable	4086	0.07	0.09	−2.69	1.51
intangible	4086	18.51	1.69	12.06	23.09
ncurrent	4086	21.09	1.52	17.17	25.29
Tobin’s Q	4086	1.91	1.14	0.88	9.67
ROA	4086	0.05	0.04	0.00	0.21
size	4086	22.06	1.32	19.07	27.00
leverage	4086	0.40	0.20	0.05	1.04
cash	4086	19.03	1.64	14.69	24.25
supervisor	4086	11.72	2.89	0.00	18.62
profit	4086	18.98	1.47	15.06	23.64
netpro	4086	18.78	1.48	14.82	23.40
subsidies	4086	16.14	1.55	11.00	19.87
**Panel B. Green Innovation of All the Enterprises in Different Years**					
**Year**	**N**	**Mean**	**sd**	**min**	**max**
2010	366	0.78	3.81	0	48
2011	355	0.98	3.98	0	39
2012	444	1.40	5.96	0	95
2013	432	1.44	6.25	0	73
2014	421	1.92	8.33	0	119
2015	515	3.10	12.72	0	154
2016	590	2.75	15.13	0	217
2017	608	2.71	15.42	0	267
2018	677	3.29	19.64	0	277
2019	689	4.00	21.60	0	359

Note: the time range is from 2010 to 2019. See Appendix A for the specific meaning of the variables. Green innovation is the sum of green invention patents and green utility model patents.

**Table 2:** Regression results of direct effects of main variables.

	(1)	(2)	(3)	(4)
Variable	Greinnov1	Greinnov2	Greinnov1	Greinnov2
SHHK	3.586 ***	1.042 ***	2.493 ***	0.578 **
	(0.922)	(0.328)	(0.493)	(0.166)
education			1.542 ***	−0.024
			(0.300)	(0.032)
sustainable			−2.188 **	−0.824 ***
			(0.759)	(0.170)
intangible			0.577	0.120
			(0.283)	(0.074)
Tobin’s Q			0.317 ***	0.153
			(0.024)	(0.091)
ROA			8.275 ***	7.007 ***
			(1.508)	(1.113)
size			2.651	0.795 **
			(1.324)	(0.286)
leverage			−3.723 **	−2.013 **
			(0.829)	(0.687)
cash			−0.055 **	0.131 *
			(0.013)	(0.047)
supervisor			0.117	0.047
			(0.094)	(0.031)
profit			−0.022	−0.224 **
			(0.347)	(0.064)
netpro			−0.198	−0.044
			(0.350)	(0.036)
subsidies			0.209 ***	0.156 ***
			(0.009)	(0.034)
ncurrent			−1.363	−0.237
			(1.014)	(0.155)
Time FE	No	No	Yes	Yes
Industry FE	No	No	Yes	Yes
Firm FE	Yes	Yes	Yes	Yes
Adj. R^2^	0.027	0.009	0.041	0.020
N	4086	4086	4086	4086

Note: Robust standard errors clustered at the industry level are in parentheses. since 2014 and 2016 are the years when the stock market liberalization took place, relevant data were deleted. ***, **, * denote statistical significance at the 1, 5, and 10% levels (two-tailed), respectively.

**Table 3:** Regression results of mechanism analysis.

	Managers’ Environment Protection Attention			Analysts’ Attention		
	(1)	(2)	(3)	(4)	(5)	(6)
Variables	Ceoatt	Greinnov1	Greinnov2	Anaatt	Greinnov1	Greinnov2
SHHK	2.215 ***	2.476 ***	0.565 **	0.884 *	2.650 **	0.668 ***
	(0.302)	(0.492)	(0.165)	(0.411)	(0.621)	(0.081)
ceoatt		0.008 ***	0.005 ***			
		(0.001)	(0.001)			
anaatt					0.070 ***	0.039 ***
					(0.009)	(0.002)
education	0.371	1.539 ***	−0.026	0.027	2.073 ***	0.143 **
	(0.440)	(0.301)	(0.032)	(0.104)	(0.418)	(0.033)
sustainable	−4.063	−2.140 **	−0.799 ***	7.889 **	−5.239 *	−2.944 *
	(3.328)	(0.722)	(0.156)	(2.172)	(2.114)	(1.149)
intangible	0.915	0.572	0.115	−0.013	0.720 *	0.170
	(0.713)	(0.277)	(0.071)	(0.154)	(0.312)	(0.104)
Tobin’s Q	−0.034	0.319 ***	0.153	1.859 ***	0.137	−0.032
	(0.199)	(0.023)	(0.091)	(0.234)	(0.112)	(0.047)
ROA	6.845	8.148 ***	6.972***	41.022 ***	12.669 **	6.641
	(4.761)	(1.493)	(1.095)	(3.015)	(2.798)	(3.287)
size	−4.097 ***	2.685	0.813 **	7.815 ***	2.872	0.231
	(0.433)	(1.332)	(0.286)	(0.726)	(1.539)	(0.201)
leverage	7.538 **	−3.774 ***	−2.054 **	−4.795 **	−4.423 ***	−1.568 **
	(2.184)	(0.810)	(0.692)	(1.188)	(0.508)	(0.427)
cash	0.204	−0.057 **	0.130 *	−0.150 **	−0.099 **	0.215 *
	(0.175)	(0.013)	(0.048)	(0.052)	(0.035)	(0.084)
supervisor	0.369 *	0.114	0.044	0.158	0.169	0.075
	(0.135)	(0.093)	(0.030)	(0.091)	(0.125)	(0.045)
profit	−2.602 **	0.002	−0.209 **	1.419 **	−0.026	0.132
	(0.577)	(0.353)	(0.061)	(0.469)	(0.391)	(0.064)
netpro	3.568 **	−0.229	−0.064	−0.015	−0.567	−0.234 **
	(0.849)	(0.359)	(0.034)	(0.237)	(0.344)	(0.079)
subsidies	−0.226	0.214 ***	0.158 **	−0.031	0.248 ***	0.250 **
	(0.223)	(0.011)	(0.035)	(0.217)	(0.024)	(0.067)
ncurrent	1.680	−1.391	−0.246	−1.139 ***	−1.930	−0.259 *
	(0.832)	(1.025)	(0.153)	(0.221)	(1.112)	(0.109)
Time FE	Yes	Yes	Yes	Yes	Yes	Yes
Industry FE	Yes	Yes	Yes	Yes	Yes	Yes
Firm FE	Yes	Yes	Yes	Yes	Yes	Yes
Adj. R^2^	0.078	0.042	0.020	0.263	0.052	0.034
N	4071	4071	4071	3088	3088	3088

Note: robust standard errors clustered at the industry level are in parentheses. ***, **, * denote statistical significance at the 1, 5, and 10% levels (two-tailed), respectively.

**Table 4:** Results of propensity score matching difference in differences (PSM-DID) method.

**Panel A. Balance Test**					
**Variables**	**Match**	**Treated Group**	**Controlled Group**	**%Bias**	***p*-Value**
education	Unmatched	3.7221	3.6776	6	0.073 *
	Matched	3.7217	3.748	−3.5	0.343
sustainable	Unmatched	0.08645	0.06352	30	0.000 ***
	Matched	0.08649	0.09005	−4.7	0.309
intangible	Unmatched	19.408	18.071	86.3	0.000 ***
	Matched	19.404	19.475	−4.6	0.251
cash	Unmatched	20.059	18.521	103.9	0.000 ***
	Matched	20.054	20.069	−1	0.807
supervisor	Unmatched	10.805	12.163	−48.7	0.000 ***
	Matched	10.805	10.481	11.6	0.002 ***
profit	Unmatched	19.988	18.497	115.1	0.000 ***
	Matched	19.984	19.974	0.7	0.859
netpro	Unmatched	19.77	18.29	112.9	0.000 ***
	Matched	19.765	19.748	1.3	0.748
subsidies	Unmatched	16.905	15.766	77.4	0.000 ***
	Matched	16.901	16.921	−1.4	0.722
**Panel B. Regression Results**					
		**(1)**		**(2)**	
**Variables**		**Greinnov1**		**Greinnov2**	
SHHK		2.305 ***		0.581 **	
		(0.456)		(0.200)	
Control variables		Yes		Yes	
Time FE		Yes		Yes	
Industry FE		Yes		Yes	
Firm FE		Yes		Yes	
Adj. R^2^		0.043		0.023	
N		3838		3818	

Note: robust standard errors clustered at the industry level are in parentheses. ***, **, * denote statistical significance at the 1, 5, and 10% levels (two-tailed), respectively.

**Table 5:** Regression results of robustness test.

	Include Samples in 2014 and 2016		Placebo Test	
	(1)	(2)	(3)	(4)
Variables	Greinnov1	Greinnov2	Greinnov1	Greinnov2
SHHK	1.846 ***	0.352 **		
	(0.360)	(0.103)		
SHHK1			0.234	0.275
			(0.175)	(0.405)
education	1.372 ***	0.028	1.319 ***	0.173
	(0.105)	(0.015)	(0.145)	(0.134)
sustainable	−1.808 *	−0.864 ***	−1.829 ***	−1.051
	(0.664)	(0.147)	(0.379)	(1.171)
intangible	0.466	0.131	0.569	0.187
	(0.266)	(0.076)	(0.350)	(0.125)
Tobin’s Q	0.186 ***	0.109	0.281 *	0.067
	(0.020)	(0.063)	(0.122)	(0.079)
ROA	8.539 **	5.067 ***	5.431	2.860
	(2.608)	(0.736)	(4.999)	(4.706)
size	2.072	0.706 *	2.256	0.560
	(1.064)	(0.289)	(1.139)	(0.353)
leverage	−3.041 ***	−1.776 **	−3.778 ***	−1.574
	(0.396)	(0.606)	(0.372)	(1.118)
cash	−0.045	0.084 *	−0.046	0.067
	(0.061)	(0.039)	(0.078)	(0.061)
supervisor	0.090	0.042	0.083	0.026
	(0.065)	(0.029)	(0.067)	(0.065)
profit	0.102	−0.120 **	0.159	0.024
	(0.307)	(0.041)	(0.364)	(0.201)
netpro	−0.232	−0.035	−0.111	−0.026
	(0.251)	(0.047)	(0.201)	(0.221)
subsidies	0.200 ***	0.139 ***	0.210 **	0.123
	(0.034)	(0.027)	(0.058)	(0.092)
ncurrent	−0.967	−0.245	−1.169	−0.244
	(1.007)	(0.142)	(1.162)	(0.363)
Time FE	Yes	Yes	Yes	Yes
Industry FE	Yes	Yes	Yes	Yes
Firm FE	Yes	Yes	Yes	Yes
Adj. R^2^	0.035	0.016	0.029	0.018
N	5097	5097	4150	4150

Note: robust standard errors clustered at the industry level are in parentheses. ***, **, * denote statistical significance at the 1, 5, and 10% levels (two-tailed), respectively.

**Table 6:** Parallel test.

	(1)	(2)
Variables	Greinnov1	Greinnov2
before4	−2.073	−0.079
	(1.519)	(0.147)
before3	−0.915	−0.055
	(0.693)	(0.120)
before2	−0.637	0.150
	(0.444)	(0.088)
after1	1.657 **	0.578 **
	(0.759)	(0.134)
after2	3.398 **	0.439 ***
	(1.571)	(0.087)
after3	1.605 **	0.425 ***
	(0.616)	(0.046)
control variables	Yes	Yes
Time FE	Yes	Yes
Industry FE	Yes	Yes
Firm FE	Yes	Yes
Adj. R^2^	0.042	0.026
N	4086	4086

Note: Data of the year when the company participated in the Stock Market Connect program are excluded. Robust standard errors clustered at the industry level are in parentheses. ***, ** denote statistical significance at the 1, 5% levels (two-tailed), respectively.

**Table 7:** Regression results of manager heterogeneity.

	Manager Heterogeneity			
	(1)	(2)	(3)	(4)
Variable	Greinnov1	Greinnov2	Greinnov1	Greinnov2
SHHK	2.327 **	0.699 **	2.467 ***	0.423 **
	(0.531)	(0.239)	(0.403)	(0.104)
overseaex	−0.285	0.395 *		
	(0.439)	(0.150)		
SHHK × overseaex	2.001 **	−1.434		
	(0.706)	(0.773)		
duality			0.292	−0.071
			(0.436)	(0.044)
SHHK × duality			0.041	0.724 *
			(1.896)	(0.282)
education	1.557 ***	−0.027	1.574 ***	−0.019
	(0.289)	(0.025)	(0.315)	(0.027)
sustainable	−2.248 **	−0.781 ***	−2.135 **	−0.843 ***
	(0.766)	(0.169)	(0.727)	(0.183)
intangible	0.584	0.115	0.573	0.121
	(0.274)	(0.077)	(0.283)	(0.073)
Tobin’s Q	0.316 ***	0.154	0.325 ***	0.153
	(0.024)	(0.089)	(0.027)	(0.094)
ROA	8.579 ***	6.842 ***	8.072 ***	6.910 ***
	(1.458)	(1.063)	(1.391)	(1.131)
size	2.688	0.776 *	2.640	0.765 *
	(1.285)	(0.300)	(1.312)	(0.292)
leverage	−3.804 ***	−1.951 **	−3.754 **	−2.002 **
	(0.802)	(0.647)	(0.828)	(0.700)
cash	−0.058 ***	0.134 *	−0.050 **	0.135 *
	(0.011)	(0.049)	(0.014)	(0.050)
supervisor	0.118	0.047	0.116	0.047
	(0.093)	(0.032)	(0.093)	(0.031)
profit	−0.011	−0.229 **	−0.031	−0.232 **
	(0.347)	(0.068)	(0.342)	(0.076)
netpro	−0.213	−0.038	−0.190	−0.035
	(0.347)	(0.038)	(0.344)	(0.041)
subsidies	0.205 ***	0.157 ***	0.213 ***	0.163 **
	(0.012)	(0.034)	(0.013)	(0.038)
ncurrent	−1.383	−0.224	−1.348	−0.243
	(0.972)	(0.172)	(1.020)	(0.166)
Constant	−44.357 **	−14.311 *	−44.308 **	−13.872 *
	(15.325)	(5.342)	(15.329)	(5.559)
Time FE	Yes	Yes	Yes	Yes
Industry FE	Yes	Yes	Yes	Yes
Firm FE	Yes	Yes	Yes	Yes
Adj. R^2^	0.042	0.020	0.041	0.020
N	4086	4086	4086	4086

Note: robust standard errors clustered at the industry level are in parentheses. ***, **, * denote statistical significance at the 1, 5, and 10% levels (two-tailed), respectively.

**Table 8:** Regression results of enterprise heterogeneity and regional openness heterogeneity.

	Enterprise Heterogeneity		Regional Openness Heterogeneity	
	(1)	(2)	(3)	(4)
Variables	Greinnov1	Greinnov2	Greinnov1	Greinnov2
SHHK	0.457	0.194	1.556 **	0.288 ***
	(0.267)	(0.206)	(0.708)	(0.047)
SOE	−1.822	−0.050		
	(0.882)	(0.228)		
SHHK × SOE	4.360 *	0.816 *		
	(1.650)	(0.466)		
SHHK × openness			3.071 *	0.951
			(1.602)	(0.500)
education	1.475 ***	−0.034	1.510 *	−0.034
	(0.298)	(0.213)	(0.814)	(0.042)
sustainable	−2.052 *	−0.812	−2.408	−0.892 ***
	(0.742)	(1.155)	(1.686)	(0.177)
intangible	0.479	0.101	0.551	0.112
	(0.249)	(0.100)	(0.346)	(0.058)
Tobin’s Q	0.306 ***	0.152	0.335	0.158
	(0.024)	(0.143)	(0.211)	(0.099)
ROA	10.038 **	7.418	8.973	7.224 ***
	(2.286)	(5.869)	(11.828)	(1.072)
size	2.830	0.823 *	2.454 *	0.734 *
	(1.498)	(0.465)	(1.352)	(0.285)
leverage	−3.397 ***	−1.959 **	−4.056 *	−2.116 **
	(0.734)	(0.958)	(2.299)	(0.732)
cash	−0.050 **	0.132 **	−0.043	0.135 *
	(0.018)	(0.063)	(0.158)	(0.054)
supervisor	0.107	0.044	0.130	0.051
	(0.092)	(0.066)	(0.159)	(0.037)
profit	0.003	−0.223	0.057	−0.200 **
	(0.300)	(0.376)	(1.378)	(0.053)
netpro	−0.267	−0.053	−0.237	−0.056
	(0.319)	(0.190)	(0.929)	(0.041)
subsidies	0.208 ***	0.156 *	0.222	0.160 ***
	(0.024)	(0.087)	(0.229)	(0.032)
ncurrent	−1.030	−0.176	−1.318	−0.223
	(0.966)	(0.394)	(1.188)	(0.165)
Constant	−51.284 *	−15.784 *	−41.354 *	−13.637 *
	(18.637)	(9.163)	(22.329)	(5.273)
Time FE	Yes	Yes	Yes	Yes
Industry FE	Yes	Yes	Yes	Yes
Firm FE	Yes	Yes	Yes	Yes
Adj. R^2^	0.051	0.021	0.045	0.021
N	4086	4086	4086	4086

Note: the coefficient of openness is omitted. robust standard errors clustered at the industry level are in parentheses. ***, **, * denote statistical significance at the 1, 5, and 10% levels (two-tailed), respectively.

## Data Availability

The data presented in this study are available on request from the corresponding author.

## Appendix A

**Table A1:** Specifications of variables.

Variable	Definition
greinnov1	Number of green invention patents of enterprises
greinnov2	Number of green utility model patents of enterprises
SHHK	If enterprise *i* is in the list of the Shanghai or Shenzhen Stock Connect in the year *t*, SHHK equals 0 before the year *t*, and 1 after the year *t*. A enterprise’s stock not listed on the Stock Connect has a value of 0 in all years.
ceoatt	Managers’ attention to environmental protection. The frequency of the key words in the report of the board of directors
anaatt	Analyst attention. In a year, how many analysts (teams) have conducted tracking analysis on the enterprise, and the number of one team is 1
SOE	If the enterprise is a state-owned enterprise, the variable is assigned to 1; otherwise, the variable is assigned to 0
overseaex	If the CEO of the enterprise has overseas study or work experience, the variable value is 1; otherwise, the variable value is 0
duality	If the chairman of the enterprise is also the CEO, the variable value is 1; otherwise, the variable value is 0
openness	If the enterprise is in the first level, its openness is high, and the variable value is 1; otherwise, the value is 0
education	The higher the score, the lower the academic background
sustainable	Sustainable growth rate. (net profit/total balance of owner’s equity at the end of the period) * [1-pre dividend per share/(net profit at the end of the period/paid in capital at the end of the period)]/(1-numerator)
intangible	Natural logarithm of net intangible assets
ncurrent	Natural logarithm of net non current assets
Tobin’s Q	Tobin’s Q
ROA	Net profit after tax/total assets
size	Natural logarithm of total assets
leverage	Total liabilities/total assets
cash	Natural logarithm of net cash flow from operating activities
supervisor	The natural logarithm of the board of supervisors
profit	Natural logarithm of total profit
netpro	Natural logarithm of net profit
subsidies	Natural logarithm of government subsidies
